# A Microfluidic Platform for the Characterisation of CNS Active Compounds

**DOI:** 10.1038/s41598-017-15950-0

**Published:** 2017-11-16

**Authors:** Christopher MacKerron, Graham Robertson, Michele Zagnoni, Trevor J. Bushell

**Affiliations:** 1 Centre for Microsystems and Photonics, Electronic and Electrical Engineering, University of Strathclyde, Glasgow, G1 1XW UK; 2 Strathclyde Institute of Pharmacy and Biomedical Sciences, University of Strathclyde, Glasgow, G4 0RE UK

## Abstract

New *in vitro* technologies that assess neuronal excitability and the derived synaptic activity within a controlled microenvironment would be beneficial for the characterisation of compounds proposed to affect central nervous system (CNS) function. Here, a microfluidic system with computer controlled compound perfusion is presented that offers a novel methodology for the pharmacological profiling of CNS acting compounds based on calcium imaging readouts. Using this system, multiple applications of the excitatory amino acid glutamate (10 nM–1 mM) elicited reproducible and reversible transient increases in intracellular calcium, allowing the generation of a concentration response curve. In addition, the system allows pharmacological investigations to be performed as evidenced by application of glutamatergic receptor antagonists, reversibly inhibiting glutamate-induced increases in intracellular calcium. Importantly, repeated glutamate applications elicited significant increases in the synaptically driven activation of the adjacent, environmentally isolated neuronal network. Therefore, the proposed new methodology will enable neuropharmacological analysis of CNS active compounds whilst simultaneously determining their effect on synaptic connectivity.

## Introduction

The burden of CNS disorders, combined with an increasingly aged population, is one of the key global health challenges of our time. Indeed, the World Health Organization has highlighted that up to 1 billion people suffer from neurological disorders and those suffering from dementia, currently exceeding 20 million, are expected to double every 20 years unless action is taken^1^. Despite global R&D pharmaceutical spending in brain disorders exceeding $140 billion per annum, and its growth set to continue^2^, early stage drug discovery has serious cost implications and high attrition rates, as investigational CNS-leads fail due to the well-known bottleneck in late stage clinical trials^3^. A recent meta-analysis on the clinical success rates of drugs in the development pipeline revealed that approximately, only 1 in 10 CNS active compounds eventually meets FDA approval^4^. The most common reasons for this level of attrition ranged from inadequate *in vitro* and *in vivo* models, to poor drug physicochemical properties and efficacy, in addition to *in vivo* toxicity^4,5^. Therefore, new technologies and methodologies for characterising CNS active compounds are a priority if healthcare and well-being are to be improved.

Early stage drug development heavily relies upon *in vitro* cultures, with neurons grown on coverslips prior to drug screening. However, when combined with plate-based high-throughput instrumentation, there is often failure in monitoring synaptic activity typical of the *in vivo* microenvironment^6,7^, as only single neuronal networks can be investigated with this approach. Standard laboratory *in vitro* techniques used to screen for CNS active compounds include electrophysiological assays, such as patch clamping and multi-electrode arrays (MEAs), as well as optical calcium (Ca^2+^) imaging techniques. Whilst the use of patch clamping provides exceptional temporal resolutions of neuronal activity in response to drug application^8–10^, it is time consuming, requires specialist training and is low throughput. Alternatively, cells can be grown across MEA substrates to obtain electrophysiological readouts across an entire neuronal network^11^, however the likelihood of a cell interfacing with a single electrode is low, and recordings may not detect subthreshold readings which would indicate if either excitatory or inhibitory signals were received^12^. The use of Ca^2+^ imaging instead allows repeated acute recordings of cellular activity without inducing toxicity, but the technique is typically constrained to low temporal resolutions. However, it provides single-cell spatial resolution over a wide cultured area and can be used to carry out cost-effective investigations of synaptic activity^13^.

The use of microfluidic procedures to model *in vitro* pathological conditions has gradually increased over the past decade, due to the level of control and manipulation available over the cellular microenvironment and experimental conditions^14,15^. These systems also show potential for use as drug discovery platforms, taking advantage of engineering techniques to facilitate high-throughput pharmacological assays, either by device design or the incorporation of perfusion systems to wash on/off multiple compounds^16–19^. Further advantages of microfluidic systems include low production costs, reduction in drug sample volumes, and the ability to analyse multiple, environmentally isolated but functionally connected cultures^20–23^, a degree of experimental capability that is lacking in conventional culturing techniques. Additionally, the transparent nature of poly-dimethylsiloxane (PDMS) based microfluidic devices make them ideal for carrying out both quantitative and qualitative pharmacological assays via Ca^2+^ imaging techniques^20,21,24^. As such, microfluidic systems are versatile tools that are well suited to perform pharmacological research^15,20,21,25–27^.

In this study, we present a microfluidic system that integrates computer-controlled perfusion of multiple compounds with Ca^2+^ imaging techniques to provide a platform for the characterisation of CNS active compounds. The novelty of this approach is its ability to simultaneously detect direct responses within a primary hippocampal culture to repeated drug applications whilst monitoring the consequent synaptic activity in an adjacent, functionally connected but environmentally isolated hippocampal culture. Hence, the proposed platform provides a novel, miniaturised solution amenable to CNS drug discovery, offering the ability to simultaneously screen both the direct effects of compounds on cells, as well as how such drugs influence communication between synaptically connected cultures.

## Materials and Methods

### Device fabrication and preparation

Microfluidic devices were fabricated using photo/soft-lithography techniques as described previously^20^, comprising of an array of microchannels between two culture chambers that are fluidically addressable via inlet/outlet wells. In brief, a two-layer microfluidic master was fabricated by spinning SU8 photoresist (3000 series, Microchem, US) onto a silicon wafer. The first layer (SU8 3010) created the microchannels (7 μm thick, 10 μm wide and 450 μm long), and the second layer (SU8 3035), the culture chambers (80 μm thick, 2 mm wide and 9.5 mm long). The silicon master was then silanized by vapour deposition of 1 H,1 H,2 H,2H-perfluorooctyl-trichlorosilane (Sigma Aldrich, UK) for 1 hour. PDMS was poured onto the master at a 10:1 ratio of base to curing agent and degassed in a vacuum desiccator prior to curing at 80 °C for 3 hours. The PDMS layer was then peeled off the master and devices were cut to the desired size, with the inlet/outlet wells (4 mm diameter) of each chamber created using a biopsy punch. Microfluidic devices were finally cleaned and irreversibly bonded to coverslips using oxygen plasma. Bonded devices were UV sterilised for 15 minutes, treated with poly-L-lysine solution (PLL, 10 μg ml^−1^) for 1.5 hours, and washed with sterile Neurobasal-A medium.

### Microfluidic perfusion protocol

**Figure 1:**
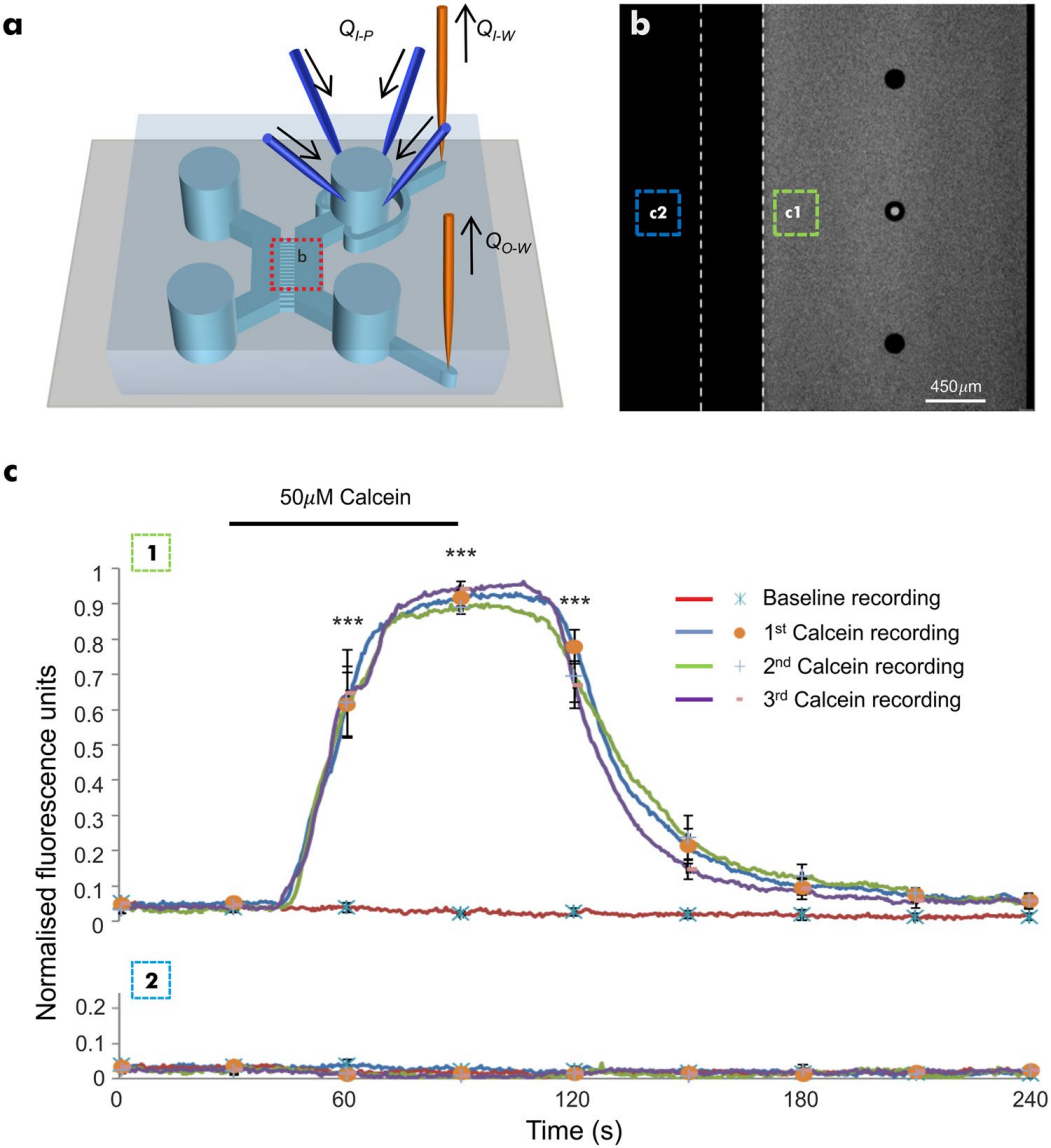
Development and characterisation of the microfluidic perfusion system. **(a)** Schematic representation of a dual chamber microfluidic device with inlet/outlet needle ports for the direct perfusion/withdrawal of fluid in/from the wells. Computer-controlled syringe pumps were used for fluid injection and withdrawal. **(b)** A representative image showing absence of cross contamination in the naïve chamber and microchannels during perfusion of calcein (50 µM) in the perfused chamber (dashed lines indicate the microchannel array separating the perfused chamber from the naïve chamber). **(c)** Fluorescent profiles in the perfused **(c1)** and naïve **(c2)** chambers, respectively upon repeated delivery of calcein solution from 3 different pumps. This demonstrated a lack of cross-contamination between the chambers and the robustness of the perfusion protocol (n = 9 devices). Data is expressed as mean ± S.E.M. (one-way ANOVA with post-hoc Tukey’s test. ^***^Denotes P < 0.001, relative to baseline readings).

Using dual culture chamber microfluidic devices, a steady flow rate in a single culture chamber (perfused chamber; with a constant flow rate *Q*_*ch*_) was achieved by creating a constant hydrostatic pressure difference between its inlet and outlet wells. The adjacent culture chamber (naïve chamber) was kept in almost static fluid conditions. Polytetrafluoroethylene (PTFE) tubing (0.255 mm inner diameter, ~700 mm length, Cole-Parmer, UK) connected glass syringes to needle ports created through the PDMS wall of the inlet-outlet wells of the perfused chamber, and the fluid was actuated using syringe pumps (AL-1000, World Precision Instruments). Up to four syringe pumps were connected to one inlet well of the device for fluid injection, with only one of these syringe pumps active at any given time and set to a constant flow rate (*Q*_*I−P*_). Two further syringe pumps were used for fluid withdrawal at constant flow rates, one connected to the inlet (*Q*_*I−W*_) and one to the outlet (*Q*_*O−W*_) well, respectively (Fig. 1a). Syringe pumps were computer-controlled via an in-house developed MATLAB (R2015a) program and ensured reliable activation/deactivation of each flow rate at the desired times. At the beginning of the assay, the following conditions were set to achieve a steady flow rate in the perfused chamber (*Q*_*Ch*_):

1 $${Q}_{I-P} > {Q}_{I-W} >  > {Q}_{O-W}$$

2 $${Q}_{I-P}-{Q}_{I-W}={Q}_{Ch}={Q}_{O-W}$$

These set of equations give rise to a self-adjusting hydrostatic pressure between the inlet and outlet wells of the perfusion chamber that reaches quickly a dynamic equilibrium. On/off alternation of the injection syringe pumps was only used to perfuse different solutions in the chamber. Due to the module of the flow rates in Eq. 1, a rapid exchange of the fluid solution in the inlet well was obtained without affecting its hydrostatic pressure and leading to a prompt injection of the new fluid solution into the chamber. The time to completely exchange a perfusate in the well was directly proportional to the inlet well’s hydraulic retention time (*HRT*), where the fluid volume of the well (*V*_*I−well*_) was replaced under a steady flow rate, resulting in *HRT* ≈ *V*_*I−well*_ / *Q*_*I−P*_ (estimated at ~25 seconds for the current setup). Therefore, for a given steady flow rate (*Q*_*Ch*_) in the perfusion chamber with a defined cross-section (*S*_*Ch*_), the time (*T*_*ex*_) taken for the full perfusate (i.e. 100% concentration) to travel a distance *L* within the chamber from the inlet well can be estimated as:

3 $${T}_{ex}\approx HRT+\frac{{S}_{Ch}\times L}{{Q}_{Ch}}$$

Under these conditions, *T*_*ex*_ is the time required for the complete exchange of solution in the inlet well and the subsequent arrival of the new solution at an arbitrary point that is a distance *L* away from the inlet well within the perfused chamber. When *L* is half the length of the culture chamber, *T*_*ex*_ is estimated at ~35 seconds for the current device geometry. To avoid cross contamination between the perfused and naïve chambers, the initial volume of fluid in the wells connecting the naïve and perfused chambers were adjusted to 50 μl/well and 10 μl/well, respectively. This established a hydrostatic pressure gradient across the array of microchannels that was maintained throughout the assay. Considering the ratio between the hydraulic resistance of the microchannel array and culture chambers^20^, the hydrostatic pressure gradient resulted in the formation of a negligible flow of vehicle from the naïve to the perfused chamber that did not influence the perfusion protocol.

The robustness of this perfusion model was validated experimentally using calcein as a fluorescent marker (Fig. 1b,c). Non-cultured microfluidic devices were connected to the perfusion setup, delivering deionised (DI) water through the culture chamber at a steady flow rate (4 μl min^−1^), with three other syringes primed with calcein (50 μM in DI water). Devices were then transferred to an inverted microscope (Axio Observer A1, Zeiss) using a 2.5× objective and the field of view was set to span an area that monitored both the microchannel array and the culture chambers. Recordings were carried out using an EMCCD camera (LucaR, Andor Technologies) at a frame rate of 2.0 Hz and an exposure time of 0.4 seconds. Time-lapsed images were obtained using Andor SOLIS.

A single region of interest (ROI) was selected for the naïve and perfused culture chamber to obtain mean fluorescent readouts on either side of the microchannel array in response to calcein perfusions. The raw data (as mean value of the ROI) obtained from individual devices was then scaled using the following equation:

4 $$f(x)=\frac{(x-\,\min )}{(\max \,-\,\min )}$$

Where *x* is the mean raw fluorescent data of each time trace/ROI (sampled at 2 Hz) and *min* and *max* are the absolute minimal and maximal mean fluorescent intensity values. Processed data from separate recordings across multiple devices were then averaged.

### Primary hippocampal culture

Primary hippocampal cultures were prepared as described previously^8,20,28^. Briefly, Sprague Dawley rat pups (1–2 days old) were killed via cervical dislocation, in accordance with the UK Home Office guidelines. The brain was then removed, hippocampi dissected out and placed in dissection solution containing (in mM) NaCl 6.78, KCl 0.40, NaHCO_3_ 2.18, NaH_2_PO_4_ 0.16, MgSO_4_ 0.15, glucose 4.5, CaCl_2_ 0.22. The hippocampi were then incubated for 20 minutes in papain solution (1.5 mg ml^−1^; Sigma Alrdich, UK) diluted in dissection solution. The tissue was then dissociated via trituration in bovine serum albumin solution (BSA; 10 mg ml^−1^ in dissecting solution; Sigma Aldrich, UK), the cell solution was then spun down at 2000 rpm for 2 minutes and re-suspended in 1 ml of supplemented Neurobasal-A medium (L-glutamine 2 mM, B27 2% v/v; Life Technologies, UK) at a concentration of 5 × 10^6^ cells ml^−1^. Cells were introduced into either one or both chambers of the device (~2.5 × 10^4^ cells per inlet well) as required and incubated for 15 minutes before inlet/outlet wells were filled with 50 μl of supplemented media. Devices remained in a humidified incubator for up to 14 days *in vitro* (DIV) prior to experimentation, with media replenished every 2–3 days.

### Assessment of perfusion effects on cell viability

Hippocampal cultures (12–14 DIV) in microfluidic devices were washed with a HEPES based external solution (HBS, containing, in mM: NaCl 140, KCl 5, MgCl_2_ 2, HEPES 10, D-glucose 10, CaCl_2_ 2; pH = 7.4; 310 mOsm). Cultured devices were connected to the perfusion setup and volumes adjusted to 50 μl and 10 μl in the wells connected to the naïve and perfused chamber, respectively. Subsequently, multiple values of steady-state flow rates (in a range of 0.4–10 μl min^−1^) were sustained in the perfused chambers for an hour and the cells were then stained for live/dead assessment. As control experiments, devices were also either left in stasis for an hour (Static t_1h_, with all wells containing 50 μl) or were stained for cell death immediately after removal from the incubator (Static t_0h_). Cultures were treated with propidium iodide (PI, 20 μM) & Hoechst (4.5 μM) made up in HBS for 30 minutes, then washed and allowed to recover for 15 minutes. Devices were then transferred to an inverted microscope (Axio Observer Z1, Zeiss), with bright-field and fluorescent images for each culture chamber captured using a 10× objective and a cooled CCD camera (AxioCam MRm, Zeiss), analysed with ZEN 2012 (Blue edition, Zeiss). Dead cells were identified by the co-staining of Hoechst and PI, whilst living cells were identified by the sole staining of Hoechst.

### Immunocytochemistry

Microfluidic hippocampal cultures (12 DIV) were fixed and stained to identify neuronal and glial populations. Briefly, using a previously established protocol^20^, cultures were washed with phosphate buffered saline solution (PBS) and fixed with ice cold paraformaldehyde (4% w/v, 10 min), followed by ice cold methanol treatment (100%, 10 min). Cultures were then washed with PBS and permeabilised with Triton-X100 (0.01%, 10 min), prior to treatment with blocking solution (in % w/v; foetal bovine serum 5, BSA 1) for 1 hour to prevent non-selective binding. Cultures were incubated at 4 °C overnight with primary antibodies β-III-Tubulin (Life Technologies, UK; neuronal specific cytoskeleton marker; 1:500 dilution), glial fibrillary acidic protein (GFAP; Sigma Aldrich, UK; astrocytic marker; 1:500 dilution), synaptophysin (Millipore, UK; synaptic vesicle marker; 1:500 dilution) and MAP2 (Sigma Aldrich, UK; somatodendritic marker; 1:500 dilution). The cells were then washed with PBS, and incubated with the relevant fluorescently labelled secondary antibodies (Alexa 488 or Alexa 555; Life Technologies, UK; 1:200 dilution) for 1 hour at room temperature. Devices were then transferred to the inverted microscope (Axio Observer Z1, Zeiss), and imaged using 10×, 20× and 63× objectives with a cooled CCD camera (AxioCam MRm, Zeiss). Image analysis was performed using ZEN 2012 (Blue edition, Zeiss).

### Calcium imaging

#### Data acquisition

Ca^2+^ imaging was performed on cultures using techniques described previously^8,20,29^. Briefly, microfluidic cultures (10–14 DIV) were loaded with the fluorescent calcium sensitive dye Fluo-4-AM (5 μM in HBS for 1 hour at room temperature) and washed with HBS (vehicle) before being transferred to an inverted microscope (Axio Observer A1, Zeiss). Devices were then connected to the perfusion setup, delivering HBS at a steady flow rate (4 μl min^−1^). Three other syringe pumps, primed with either glutamate (0.01–1 mM), glutamate antagonists (NBQX 20 μM, DL-AP5 100 μM, (R,S)-MCPG 500 μM) or glutamate (3 μM) in the presence of glutamate antagonists, were used to perfused drugs of interest at a steady flow rate of 4 μl min^−1^. Syringe pumps were primed with glutamate (3 μM), the Na^+^ channel blocker tetrodotoxin (TTX, 1 μM) or glutamate in the presence of TTX and the solutions were perfused at a steady flow rate of 4 μl min^−1^. Devices used to generate a glutamate concentration response curve were randomly assigned three separate glutamate concentrations that were perfused in order of increasing concentration. Simultaneous recordings of Ca^2+^ transients in the two environmentally isolated, independent cultures were obtained with a cooled EMCCD camera (LucaR, Andor Technologies) using either a 5× or 10× objective, at a frame rate of 2.0 Hz and exposure time of 0.4 s. L-glutamic acid (Tocris Bioscience, UK), the glutamate receptor antagonists (NBQX, DL-AP5 & (R,S)-MCPG; Abcam, UK) and tetrodotoxin (TTX citrate; Abcam, UK) were made up as 1000× stock solutions and diluted to final concentrations in vehicle solution on the day of experimentation. Time-lapse images were processed and analysed using Andor SOLIS, MATLAB (R2015a), and BioGraph (V2.5.6; J. Dempster, University of Strathclyde). As is standard, glutamate receptor antagonists or TTX were perfused during the resting period following the first glutamate perfusion and during the subsequent glutamate application to determine their effects on glutamate-induced Ca^2+^ transients. At the end of each experiment, the perfusion was stopped and KCl (25 mM final concentration) was applied to each chamber. Cells that did not elicit an immediate and sustained increase in fluorescence were excluded from analysis (see Supplementary Fig. S1).

#### Analysis

Regions of interest (ROI) were obtained from individual cell somas and the mean fluorescent intensity per ROI per frame was used to create a graphic readout of neuronal Ca^2+^ activity, with each cell’s response trace normalised to the basal value for each recording. To produce a concentration response curve, the mean peak magnitudes obtained from increasing glutamate concentrations were normalised to the mean peak magnitude obtained from the highest glutamate application (1 mM) and the concentration response curve generated using the Hill equation:

5 $$y=\frac{{R}_{Max}}{1+{(\frac{x}{E{C}_{50}})}^{n}},$$

where *RMax* = maximum response observed, *EC*_50_ = effective concentration at which 50% maximum response is obtained, and *n* = Hill coefficient. Otherwise, changes in neuronal activity in response to indirect stimulation from the directly perfused neural network were identified by the automated counting of Ca^2+^ events. The signals of each neuron obtained over the course of the assay were stitched together, filtered (moving average filter, window size 11) and normalised to the first 24 frames of the baseline recording. Events were counted on the basis of significant increases in fluorescence exceeding 7 standard deviations of the median baseline fluorescence, occurring within a moving window of 10 seconds. This provided the number of events taking place per neuron during both the basal period and glutamate perfusion of each recording, with results normalised to time and the indirect activity expressed as events/neuron/minute (ENM).

### Statistics

All data is presented as mean ± S.E.M. using bar/scatter graphs and results compared using paired student’s t-tests, unpaired t-tests or one-way ANOVA with Tukey’s post-hoc comparison as appropriate, with differences considered significant when P < 0.05, or treated otherwise as non-significant (ns).

## Results

### A robust model for pharmacological perfusion assays

To assess the reproducibility of consecutive drug applications using the proposed system, initial experiments were performed using calcein to visualise perfusion performance and results confirmed a close match with the predicted flow behaviour (equations 1–3). The protocol developed produced a stable fluorescent plateau for the intended duration of perfusion (~30 seconds), with increasing (~35 seconds) and decreasing (~45 seconds) transients for all experiments (Fig. 1). Importantly, the absence of cross contamination between the two culture chambers was experimentally confirmed, demonstrating their fluidic isolation.

**Figure 2:**
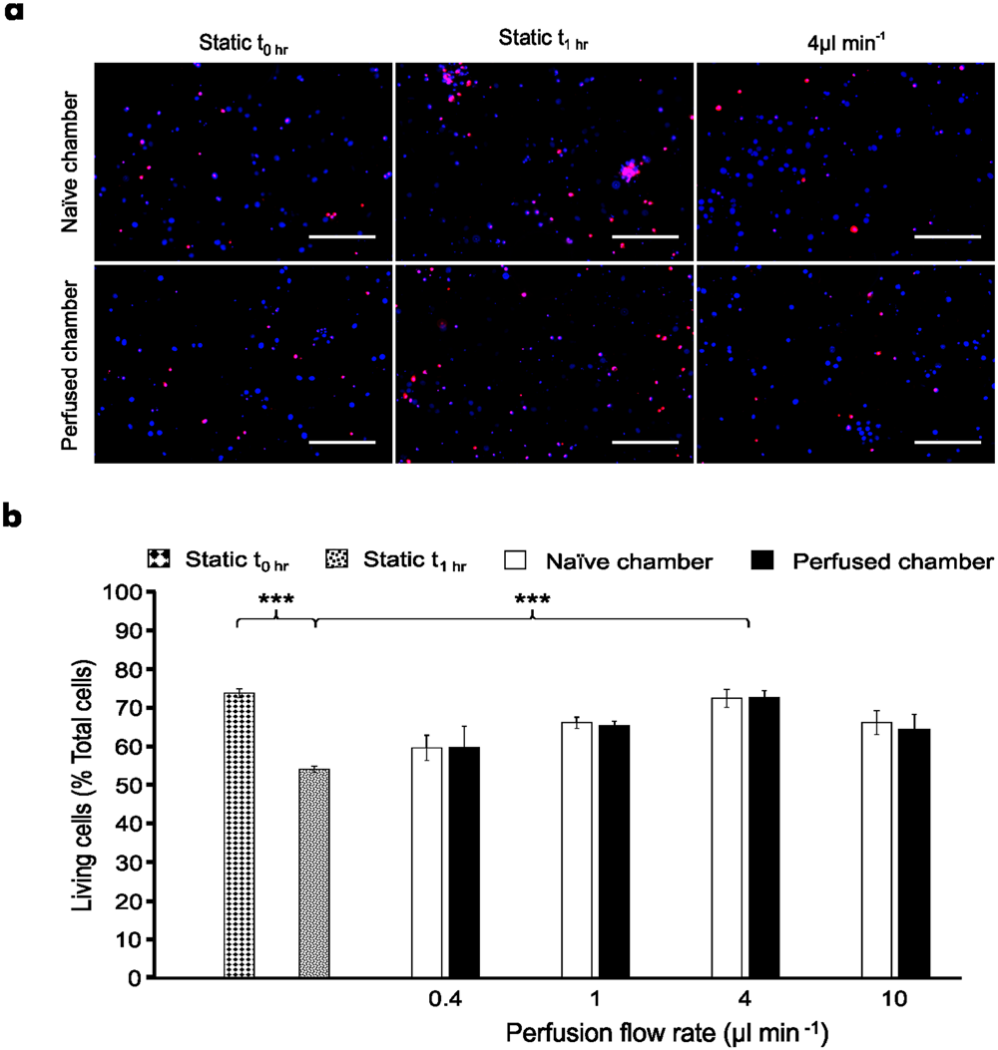
Continuous perfusion maintains cell viability between environmentally isolated neural networks. **(a)** Representative images obtained from perfused and naïve cultures which were stained with Hoechst (blue) and PI (red) following continuous perfusion experiments for 1 hour. Scale bar = 200 µm. **(b)** Microfluidic neural network viability increases in the presence of perfusion with respect to static condition after 1 hour, with an optimal flow rate of 4 μl min^−1^. Data are presented as mean ± S.E.M. with one-way ANOVA with post-hoc Tukey’s test performed; n = 21 devices from 4 cultures; ^***^denotes P < 0.001.

Having validated the robustness of the perfusion protocol, the effect of shear stress on the health of perfused hippocampal cultures (12–14 DIV) was examined (Fig. 2) to determine an optimal flow rate for the pharmacological study. As a benchmark, live/dead imaging was carried out on microfluidic cultures immediately after being removed from the incubator, with live cells accounting for 73.8 ± 0.01% (n = 3 devices, each device from a separate culture) of the total cell population (Fig. 2b). However, this decreased to 54.1 ± 0.9% (n = 6 devices from 3 cultures; P < 0.001, static t_0h_ *vs* static t_1h_) of the total cell population when the devices were left at room temperature under static conditions for an hour. In contrast, constant perfusion at room temperature, with flow rates ranging from 0.4–10 μl min^−1^ (n = 3 devices, each device from a separate culture), revealed varying effects on cell viability after an hour, with live cells accounting for 72.6 ± 1.6% (P < 0.001 *vs* static t_1h_) of the total cell population at the optimal flow rate of 4 μl min^−1^ (Fig. 2b). Furthermore, the health of the directly perfused cultures was closely matched by that of the non-perfused naïve culture across all experimental conditions (non-significant, P > 0.05). Given these results, a flow rate of 4 μl min^−1^ was used for the remainder of this study. Finally, microfluidic cultures were stained for β-III-Tubulin, GFAP and synaptophysin revealing that only neuronal, but not astrocytic processes, were able to traverse across the microchannel array to the adjacent chamber and that synapses were formed both within and outside the microchannels (see Supplementary Fig. S2). Collectively, continuous perfusion experiments and immunocytochemistry imaging indicate that synaptically driven functional communication exists between the two hippocampal cultures, as previously reported^20^.

### Perfusion permits the characterisation of CNS active compounds

**Figure 3:**
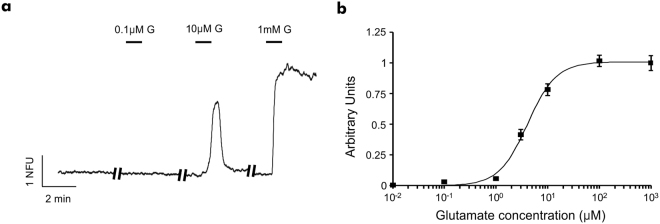
Microfluidic hippocampal cultures respond to direct glutamate (G) application in a concentration-dependent manner. **(a)** Ca^2+^ imaging trace representative of a single neuron’s response to perfusion of consecutive, increasing concentrations of glutamate (10–14 DIV). NFU = Normalised fluorescent unit. **(b)** Concentration response curve for glutamate (10 nm–1 mM) reveals an EC_50_ = 4.7 ± 0.6 μM. Data is presented as mean ± S.E.M (n ≥ 50 neurons per concentration point, curve obtained from 14 devices from 6 cultures).

As proof-of-concept work, to demonstrate the system’s ability to carry out pharmacological characterisation of neuroactive compounds, a concentration response curve was generated using the excitatory amino acid, glutamate. Perfusion of glutamate (10 nM – 1 mM) elicited reversible concentration-dependent increases in intracellular Ca^2+^ (Fig. 3a), resulting in a concentration response curve with an EC_50_ of 4.7 ± 0.6 μM (Fig. 3b).

**Figure 4:**
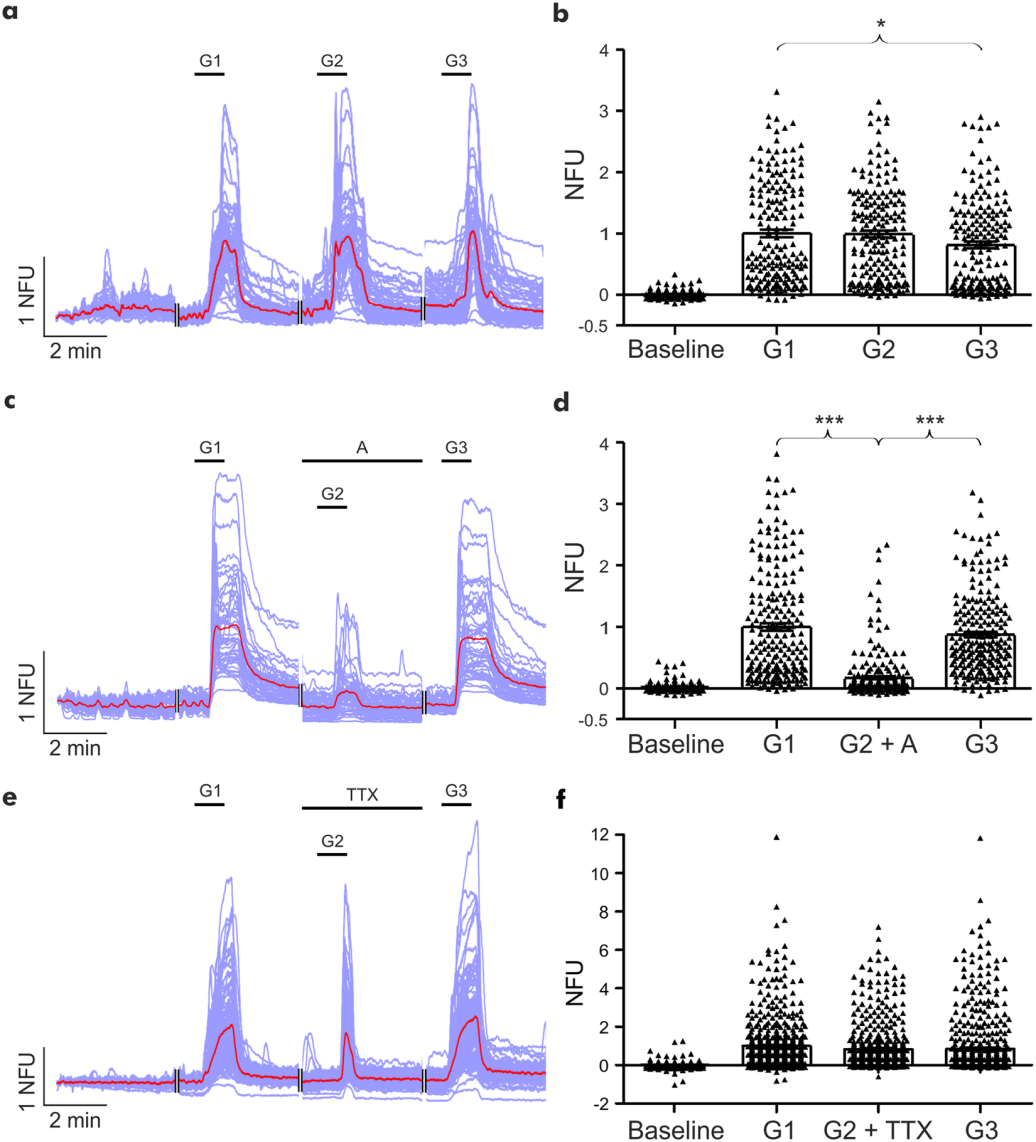
Reproducible glutamate responses are inhibited by glutamate antagonists. **(a**, **c + e)** Representative Ca^2+^ imaging traces of neuronal responses to repeated glutamate applications in the absence or presence of antagonists or TTX (purple trace: individual neuronal responses; red trace: average response). NFU = Normalised fluorescent unit. **(b)** Repeated applications of glutamate (3 μM) revealed a small but significant reduction in neuronal response during the third glutamate application. **(d)** The neuronal response to glutamate (3 μM) application was reversibly inhibited in the presence of glutamatergic antagonists (NBQX 20 μM, DL-AP5 100 μM & (R,S)-MCPG 500 μM). **(f)** The neuronal response to glutamate (3 μM) application was not significantly affected in the presence of TTX (1 μM). Data are presented as mean ± S.E.M. with one-way ANOVA with post-hoc Tukey’s test performed; n ≥ 189 neurons per application, from ≥3 devices from ≥3 separate cultures; ^*^denotes P < 0.05 and ^***^denotes P < 0.001.

Having established that our integrated perfusion system allows the generation of concentration response curves, we tested the platforms capability for the pharmacological screening of compounds. To this end, we investigated the actions of ionotropic and metabotropic glutamate receptor antagonists NBQX (20 μM), DL-AP5 (100 μM) and (R,S)-MCPG (500 μM) on glutamate (3 μM)-induced changes in intracellular Ca^2+^. Repeated glutamate perfusions elicited robust and reproducible increases in intracellular Ca^2+^ (n = 182 neurons, 4 devices, each device from a separate culture; Fig. 4a & b), which were inhibited in the presence of the glutamate antagonists to 17 ± 2.5% of the response elicited by glutamate alone (n = 219 neurons, 4 devices, each device from a separate culture; P < 0.001; Fig. 4c & d). In contrast, no significant difference in glutamate-induced increases of intracellular Ca^2+^ (n = 551 neurons, 3 devices, each device from a separate culture; Fig. 4e & f) were observed in the presence of TTX (1 μM).

### Modulation of neuronal activity in synaptically connected hippocampal cultures

**Figure 5:**
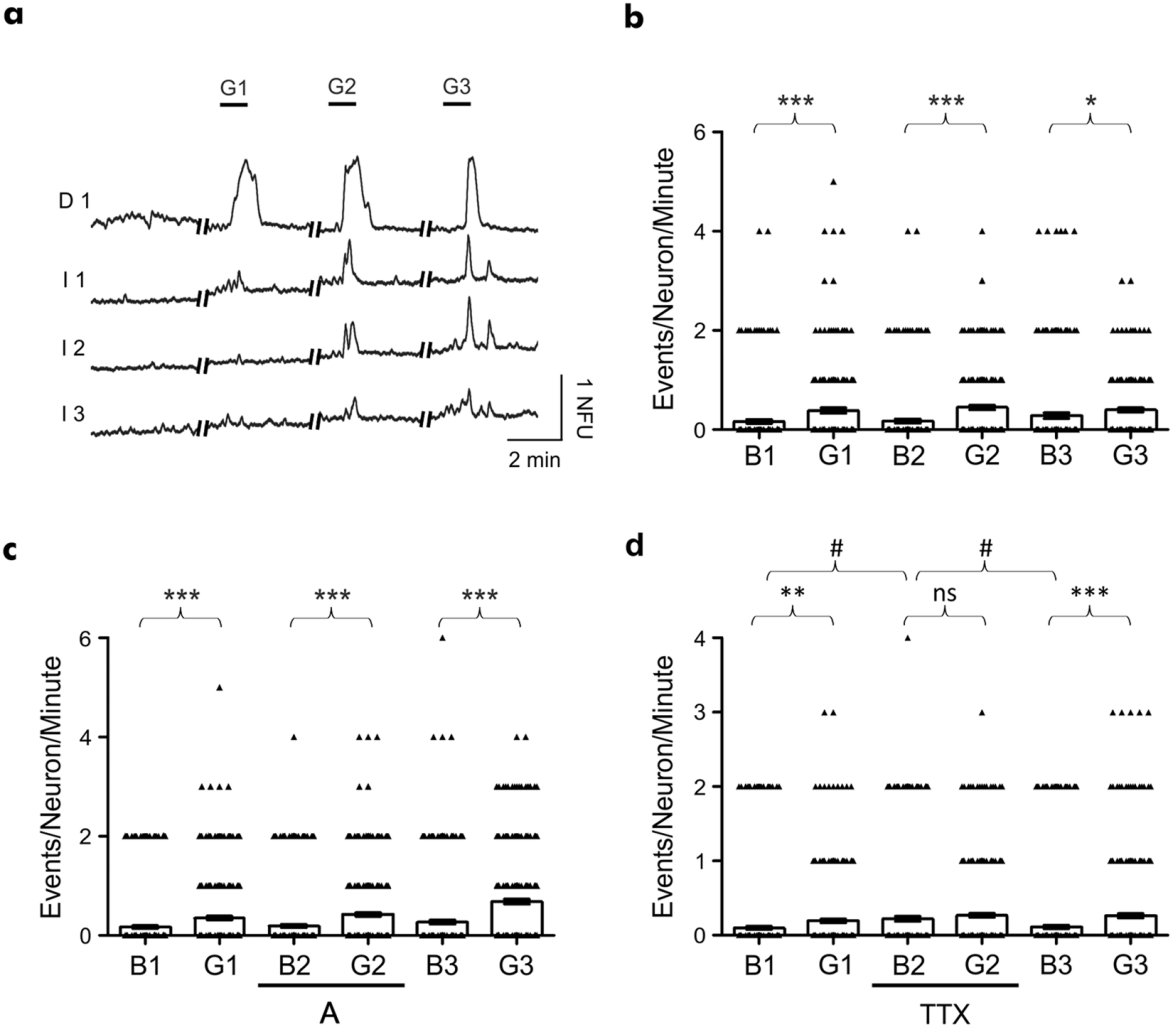
Repeated glutamate application induces increased neuronal activity in synaptically connected hippocampal cultures. **(a)** Ca^2+^ imaging traces representative of activity obtained from individual neurons in the directly perfused (trace D1) and naïve (traces I1–I3) culture chambers, demonstrating functional synaptic communication between independent hippocampal cultures. **(b)** Significant increases in the number of neuronal calcium events were observed in the naïve chamber, with respect to the basal (B) activity, in response to the three glutamate (G) applications in the perfused chamber. **(c)** Significant increases in the number of neuronal calcium events were observed in the naïve chamber, with respect to the basal (B) activity, in response to glutamate applications (G) in the absence and presence of glutamate antagonists in the perfused chamber. **(d)** Basal activity in the naïve chamber was significantly increased in the presence of TTX in the perfused chamber. Further changes in the number of neuronal calcium events were not observed following glutamate (G) application in the presence of TTX. Data are presented as mean ± S.E.M. with paired student’s t-test performed; n =  ≥ 222 neurons per application, ≥3 devices from ≥3 separate cultures; ns denotes P > 0.05, ^*^denotes P < 0.05, ^**^denotes P < 0.01, ^***^denotes P < 0.001 and ^#^denotes P < 0.05.

Having previously demonstrated functional synaptic connectivity across two environmentally isolated hippocampal cultures following a single drug application^20^, here changes in postsynaptic responses in the naïve chamber were examined as a consequence of repeated glutamate (3 μM) applications in the directly perfused chamber (Fig. 5, see Supplementary Video S1).

During the first glutamate application, neuronal activity within the naïve chamber significantly increased from a baseline of 0.16 ± 0.04 ENM to 0.38 ± 0.04 ENM (P < 0.001; n = 222 neurons; Fig. 5b). Consistent with this, the second glutamate application also induced a significant increase in neuronal activity from 0.17 ± 0.04 ENM to 0.44 ± 0.04 ENM during glutamate application (P < 0.001). Similarly, the final glutamate application elicited a significant increase in activity, from 0.27 ± 0.05 ENM to 0.4 ± 0.05 ENM (P > 0.05). In the presence of glutamate antagonists in the perfused chamber, neuronal activity was unaltered in the naïve chamber (Fig. 5c). A significant elevation in neuronal activity in the naïve chamber was observed following glutamate application in the presence of glutamate antagonists, from a baseline of 0.19 ± 0.03 ENM to 0.42 ± 0.04 ENM (P < 0.001; n = 350 neurons; Fig. 5c. In contrast, baseline activity in the naïve chamber was significantly increased from 0.10 ± 0.02 ENM to 0.22 ± 0.03 ENM (P < 0.01; B1 *vs* B2; n = 381 neurons; Fig. 5d) with the presence of TTX in the directly perfused chamber, whereas glutamate application in the presence of TTX did not induce any further increases in neuronal activity in the naïve chamber (Fig. 5d). Finally, in order to confirm that changes in neuronal activity were unidirectional, hippocampal cultures were grown in one chamber only and glutamate perfused in the non-cultured chamber. Repeated application of glutamate (3 μM) in the non-cultured chamber resulted in no changes in neuronal activity in the cultured chamber (see Supplementary Fig. S3).

## Discussion

This study presents the first successful integration of microfluidic perfusion with Ca^2+^ imaging techniques into a platform that allows the pharmacological characterisation of CNS active compounds using primary hippocampal cultures. The novelty of this approach is in examining simultaneously both the direct response to drug application (i.e. neuronal excitability) and the consequent alterations in neuronal activity in synaptically connected but environmentally isolated primary hippocampal cultures (i.e. induced synaptic activity), over customisable and repeatable conditions.

In all the experiments, the diffusive transport of compounds could be neglected with respect to their convective transport in the perfused chamber and microchannel array, as the Péclet number^30^ was always >100 and the Reynolds number was always <1. Absence of cross-contamination between chambers was experimentally confirmed using fluorescent imaging and the results were in line with previously established models^20,31–33^. Importantly, interchangeable perfusion of different solutions through a single culture chamber was achieved without disturbance in the flow. This was obtained by rapidly replacing the liquid volume inside the inlet open well of the perfusion chamber, selecting flow rate values that were much greater than that created in the perfused chamber. The open well system, in combination with carefully selected values of the flow rates (equations 1–2), generated a self-adjusting hydrostatic pressure difference between inlet and outlet wells of the perfused chamber that maintained a constant flow rate in the perfused chambers for the duration of the experiment. If the flow rate assigned to the inlet well had been the same as that of the perfused chamber, then the time taken to completely change perfusates would be significantly longer (~5 minutes), which highlights the need for the faster flow rate at the inlet well. This approach enabled a quick fluid transition without creating a pulsatile flow when exchanging buffer and drug solutions. Further improvements to the platform could be made by replacing the syringe pump setup with a pressure-driven, valve-controlled perfusion system, thus extending the number of drugs that can be tested in the system, as well as reducing the size and number of external instrumentation needed for active flow control.

Microfluidic perfusion has been extensively applied to a variety of cell types and culture conditions, including both brain slices and tissue preparations^18,34–38^. A previous approach has been reported where a single perfusion pump was used to exert a negative pressure across an isolated axonal compartment, consequently withdrawing drug solution (applied *in situ*) from a reservoir to modulate synaptic activity and protein expression^37^. The main limitations of that setup were the requirement for regular, manual drug application throughout the experimental procedure and that the flow of perfusate was not selectively applied to an environmentally isolated culture. Alternatively, a model for perfusion has also been described in which neural networks were continuously perfused with automated pumps for up to 70 days^38^, with the cells grown on a microelectrode array within a microscope onstage incubator. Whilst constant simultaneous morphological imaging and electrophysiological readout of neuronal activity was monitored over weeks, providing exceptional spatiotemporal resolutions, the protocol is time and resource heavy. Interestingly, these reports have shown that continuous perfusion alters synaptic activity and may be beneficial to the health of primary neuronal cultures^34–36^. Previous reports have shown how 2D *vs* 3D cultures, co-culture conditions and shear stress may influence gene expression, production/transport/removal of nutrients and molecular secretions^39–42^. Our findings suggest that an optimal steady flow rate confers neuroprotective properties not only to a single perfused primary culture, but also to an adjacent, synaptically connected culture. Recently, we have revealed evidence of neuroprotection against spreading toxicity between neighbouring synaptically connected but environmentally isolated hippocampal cultures^21^, and previous studies have proposed suppression of excitotoxicity via interneurons in response to mechanically-induced astrocytic release of ATP^43–45^. However, the exact mechanisms underlying this observation in the present study remain unclear.

Ca^2+^ imaging is a powerful and cost-effective technique that can be used to provide optical readouts of both spontaneous and induced cellular activity^8,29,46^. When combined with the precise spatial and temporal fluid flow control achieved using microfluidic systems, high-throughput and miniaturised assays can be performed. Whilst neuropharmacological studies have used Ca^2+^ imaging to perform high-throughput pharmacological analysis^47–50^, they are either limited in their fine control of stimulus delivery or often require user intervention to perform *in situ* drug delivery. Furthermore, such conventional systems, which integrate Ca^2+^ imaging techniques to observe neuronal excitability following compound delivery, lack neighbouring environmentally isolated cultures, thus limiting the understanding of a drug’s effect on the synaptic connectivity between cultures.

In the present study, we demonstrate proof-of-concept analysis of both neuronal excitability and consequent synaptic connectivity. First, a concentration response curve was generated by obtaining reproducible responses to a range of glutamate concentrations, revealing an EC_50_ of 4.7 μM, which compares well to that reported in the literature^51–53^. Additionally, the neuronal response to glutamate perfusion in the absence or presence of glutamatergic receptor antagonists was examined, revealing reversible inhibition of glutamate-induced transient increases in intracellular Ca^2+^. These assays highlight that the microfluidic perfusion platform described here can be utilised for the pharmacological profiling of CNS active compounds. Furthermore, our miniaturised system shows for the first time, repeatable assessment of induced network synaptic activity in addition to pharmacological profiling of directly applied compounds in a microfluidic format. Indeed, neuronal activity in the naïve hippocampal culture was increased in response to multiple glutamate applications but, strikingly, this led to an increased level of basal neuronal activity prior to the final glutamate application. Whilst the final glutamate application still induced an increase in neuronal activity with respect to baseline, repeated synaptically driven signalling appears to induce an excited state of activity in the naïve culture, which gradually becomes resistant to further stimulation. This observation mimics the increased responsiveness of neurons that undergo synaptic plasticity^54^ and in this case, increased responsiveness similar to that observed in long term potentiation (LTP). Whilst we have not probed the underlying mechanisms in the present study, these results are in line with previous studies in which LTP has been investigated in cultured hippocampal neurons using both electrophysiological and Ca^2+^ imaging techniques^55–57^. Basal neuronal activity within the naïve chamber was unaltered in the presence of glutamate antagonists whereas increases were observed following glutamate application in their presence. Given that glutamate-induced Ca^2+^ responses in the perfused chamber were significantly reduced, but not abolished, in the presence of the antagonists, the increases in the naïve chamber are likely to be induced via the residual responses observed in the perfused chamber. In contrast, basal activity in the naïve chamber was significantly enhanced in the presence of TTX in the perfused chamber. Given that our experiments, in which cultures were grown in one chamber only, revealed that the glutamate-induced increase in neuronal activity is unidirectional, this result indicates that TTX application in the perfused chamber may be impairing an inhibitory tone that controls neuronal activity within the naïve chamber. As primary hippocampal cultures grown under our experimental conditions contain approximately 6% GABAergic neurons^58^, these will exert a strong inhibitory control over excitatory neurotransmission within the cultures. Indeed, application of GABAergic antagonists has been used to mimic epileptiform-like activity in hippocampal cultures^59,60^, hence the inhibition of GABAergic neuronal firing by TTX in the perfused chamber would result in the increased activity in the naïve chamber due to decreased GABAergic tone extending from the perfused to the naïve chamber. Furthermore, glutamate induced increases in neuronal activity were abolished during the co-application of glutamate and TTX, despite increases in intracellular calcium in neurons within the perfused chamber. Collectively, these results demonstrate that changes in neuronal activity within the naïve culture chamber are driven by functional, synaptic communication from neurons in the perfused chamber.

## Conclusions

We have developed a novel platform that integrates microfluidic perfusion and Ca^2+^ imaging techniques for studying neuromodulation and synaptic connectivity between primary hippocampal cultures. We show that both direct neuronal excitability and the consequent synaptic communication between synaptically connected but environmentally isolated cultures can be simultaneously monitored, providing hundreds of readouts from each device. In addition, with the recent surge in interest in the use of human stem cell derived neurons to investigate CNS disorders^61,62^, the platform described in the present study may offer new avenues to examine the underlying causes of CNS disease and allow potential novel therapeutics to be tested in a cost-effective, miniaturised manner.

### Data availability

Access to all data underpinning this publication is restricted to a request only basis. More information and contact details are available from the University of Strathclyde at 10.15129/5a018c9b-9458-4e3f-8a92-23657abcd44e. 

## Electronic supplementary material

Supplementary information

Video 1
