# Supplementary material for: A Microfluidic Platform for the Characterisation of CNS Active Compounds
Source: Sci Rep. 2017 Nov 16;7:15692. doi: 10.1038/s41598-017-15950-0 (PMC5691080; doi:10.1038/s41598-017-15950-0)
Supplement: Supplementary file 1 — Supplementary information [file 41598_2017_15950_MOESM1_ESM.doc]

Supplementary Information

A Microfluidic Platform for the Characterisation of CNS Active Compounds

Christopher MacKerron^1^, Graham Robertson^1^, Michele Zagnoni*^1^ and Trevor J. Bushell^2^
^1^ Centre for Microsystems and Photonics, Electronic and Electrical Engineering, University of Strathclyde, Glasgow, G1 1XW, UK.
^2^ Strathclyde Institute of Pharmacy and Biomedical Sciences, University of Strathclyde, Glasgow, G4 0RE, UK
* Corresponding author: [michele.zagnoni@strath.ac.uk](mailto:michele.zagnoni@strath.ac.uk)

**Supplementary Video S1:** Calcium imaging of directly and indirectly stimulated neurons in response to repeated glutamate application in the microfluidic device.

The video shows the fluorescent readout (right panel) and the temporal traces (left panel) of representative calcium (Ca^2+^) imaging responses of neurons from data in Fig 5a. Following glutamate perfusion (three glutamate applications separated by a recovery phase), an increase in fluorescence was observed in directly stimulated neurons (D – perfused chamber) that induced Ca^2+^ events in the environmentally isolated but synaptically connected neurons in the adjacent chamber for each perfusion step (I_1_, I_2_, I_3_ – naïve chamber).

**Supplementary Figure S1:** Identification of neurons following KCl application.

**
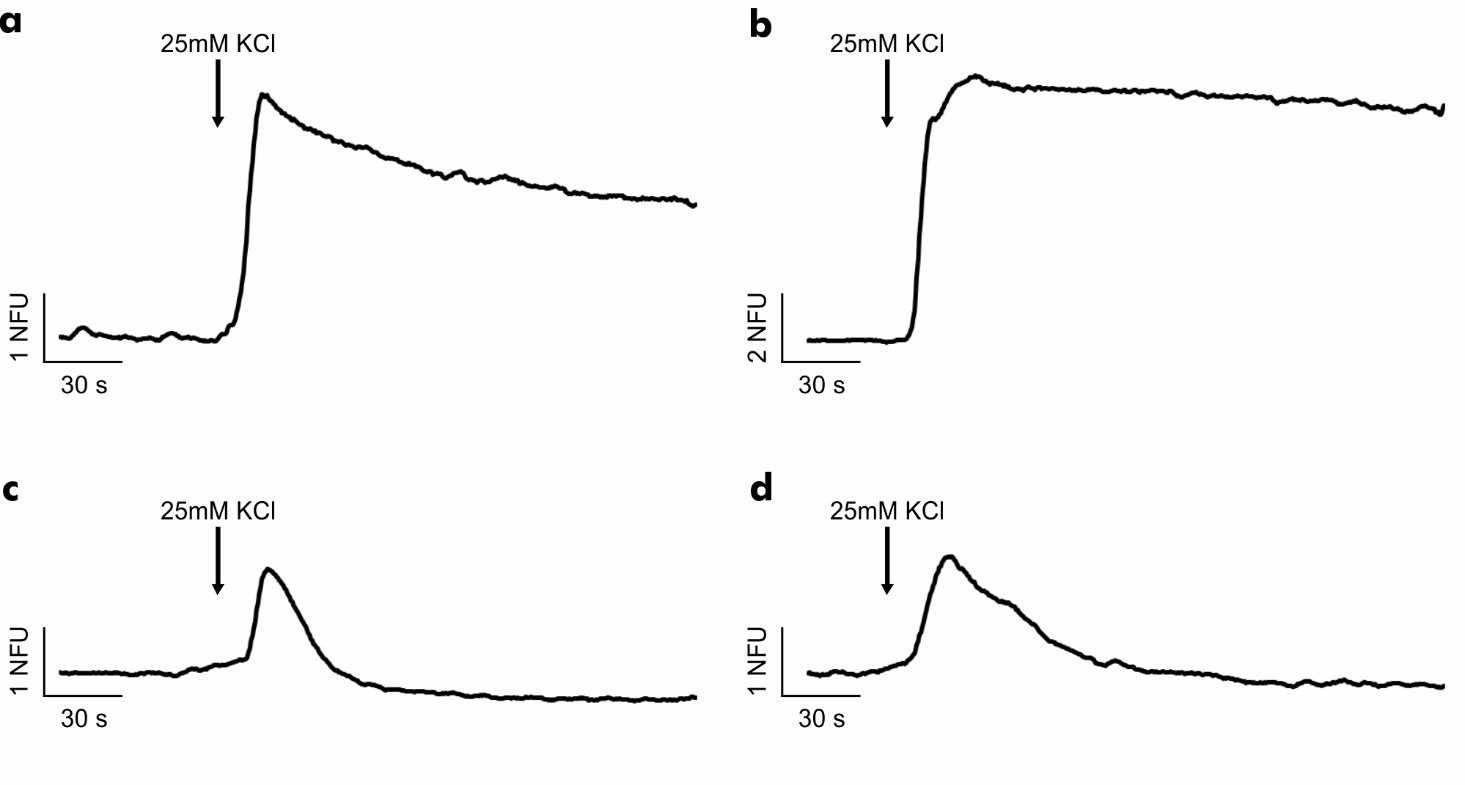
**

Following the cessation of perfusion, a final recording was initiated and KCl (25mM final concentration) was applied to both the naïve and perfused chambers for the identification of neurons. **(a, b)** Representative traces depicting neurons responding to KCl from the perfused and naïve culture chambers, respectively, displaying a sharp increase in fluorescence with minimal recovering over time. **(c, d)** Representative traces of cells from the perfused and naïve culture chambers respectively that did not elicit typical neuronal characteristics following KCl application, displaying small increases in fluorescence and recovering to basal fluorescence. These cells were thus excluded from analysis.

**Supplementary Figure S2:** Immunocytochemical staining of synaptically connected, mixed primary hippocampal networks.

**
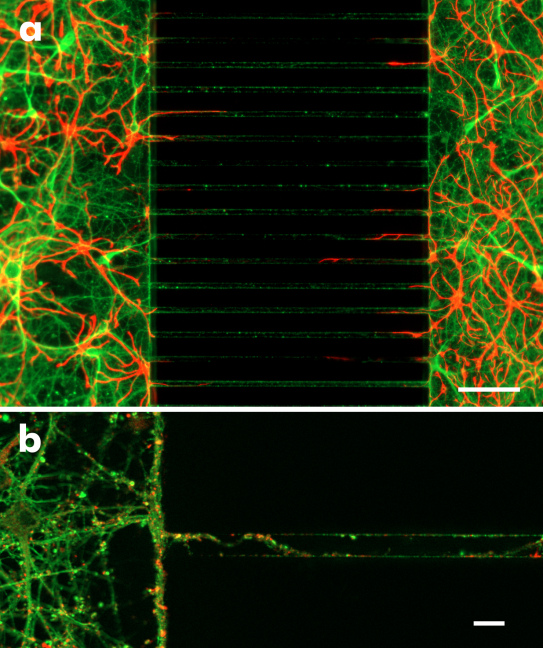
**

**a)** Primary hippocampal cultures were stained for β III-tubulin (green) & glial fibrillary acidic protein (GFAP) (red), showing that neuronal projections are able to traverse the length of the microchannels, whereas astrocyte projections cannot. **(b)** Co-staining neuronal networks for β III-tubulin (green) & synaptophysin (red) reveals the presence of synaptic vesicles on neuronal projections within the microchannel array. Scale bars = 100 µm **(a)**, 20 µm **(b)**.

**Supplementary Figure S3:** Axons exposed to repeated glutamate perfusions do not increase neuronal network activity.


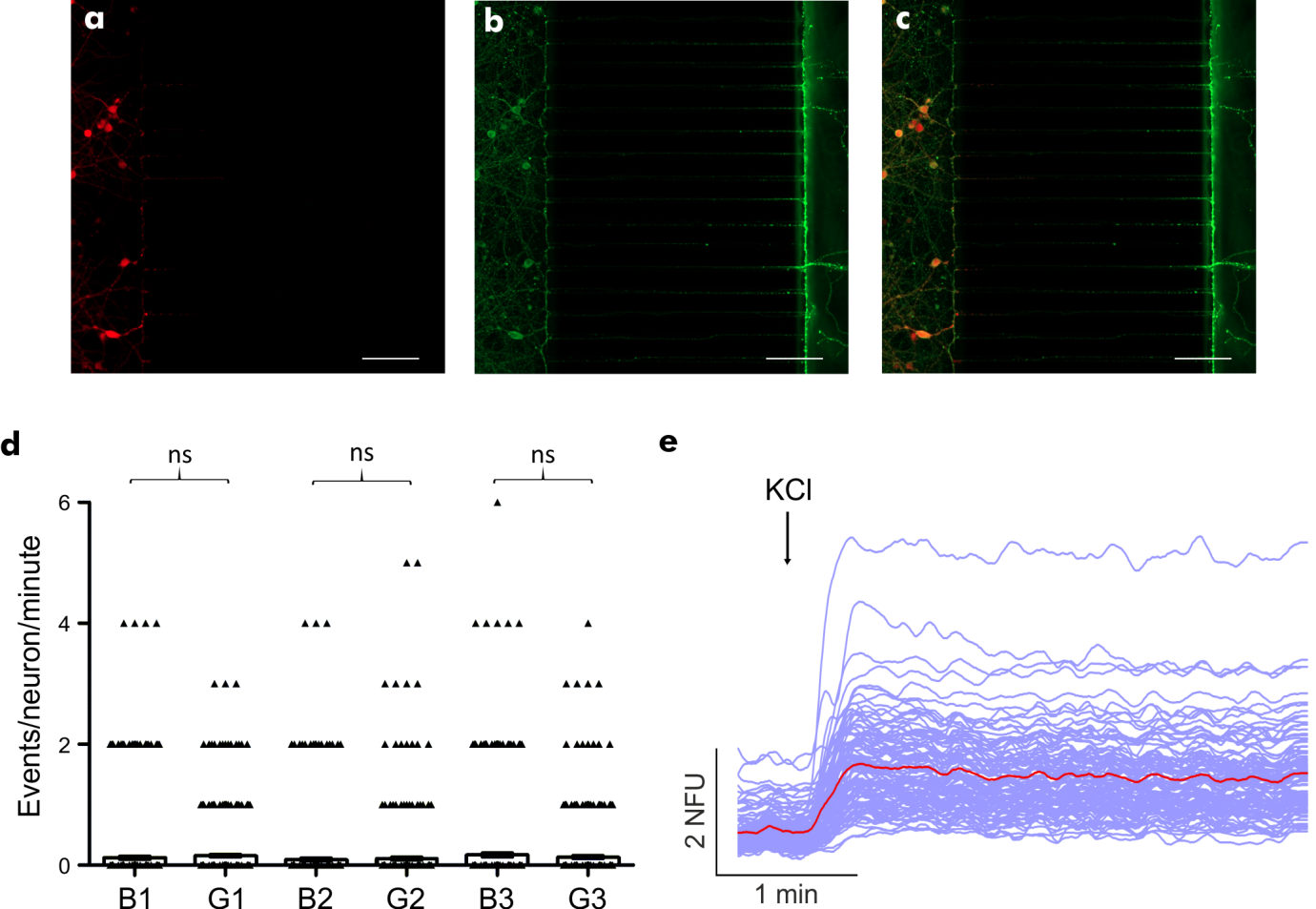


**(a, b, c)** MAP2 (red), β III-Tubulin (green) and merged images (respectively) of a single neuronal network grown in the microfluidic perfusion device, with axonal projections traversing the microchannel array into the adjacent chamber, whilst dendritic projections do not. **(d)** No significant increase in naïve network activity was observed during glutamate (3 μM) applications in the perfused chamber (containing only axons), when compared to basal activity for each respective recording. **(e)** Following the cessation of the perfusion assay, KCl (25 μM final concentration) was applied to the naïve network to reveal functional neurons. Data are presented as mean ± S.E.M. (paired student’s t-tets); n = 469 neurons per application, from 3 devices from 3 separate cultures; ns denotes P > 0.05. Scale bars = 100 µm.
